# Rapid and economical drug resistance profiling with Nanopore MinION for clinical specimens with low bacillary burden of *Mycobacterium tuberculosis*

**DOI:** 10.1186/s13104-020-05287-9

**Published:** 2020-09-18

**Authors:** Wai Sing Chan, Chun Hang Au, Yvonne Chung, Henry Chi Ming Leung, Dona N. Ho, Elaine Yue Ling Wong, Tak Wah Lam, Tsun Leung Chan, Edmond Shiu Kwan Ma, Bone Siu Fai Tang

**Affiliations:** 1 Department of Pathology, Hong Kong Sanatorium & Hospital, Hong Kong, China; 2 Department of Computer Science, The University of Hong Kong, Hong Kong, China; 3 L3 Bioinformatics Limited, Hong Kong, China

**Keywords:** Antibiotic resistance, Illumina MiSeq, MDR-TB, Nanopore MinION, NGS, Tuberculosis, XDR-TB, Xpert MTB/RIF

## Abstract

**Objective:**

We designed and tested a Nanopore sequencing panel for direct tuberculosis drug resistance profiling. The panel targeted 10 resistance-associated loci. We assessed the feasibility of amplifying and sequencing these loci from 23 clinical specimens with low bacillary burden.

**Results:**

At least 8 loci were successfully amplified from the majority for predicting first- and second-line drug resistance (14/23, 60.87%), and the 12 specimens yielding all 10 targets were sequenced with Nanopore MinION and Illumina MiSeq. MinION sequencing data was corrected by Nanopolish and recurrent variants were filtered. A total of 67,082 bases across all consensus sequences were analyzed, with 67,019 bases called by both MinION and MiSeq as wildtype. For the 41 single nucleotide variants (SNVs) called by MiSeq with 100% variant allelic frequency (VAF), 39 (95.1%) were called by MinION. For the 22 mixed bases called by MiSeq, a SNV with the highest VAF (70%) was called by MinION. With short assay time, reasonable reagent cost as well as continuously improving sequencing chemistry and signal correction pipelines, this Nanopore method can be a viable option for direct tuberculosis drug resistance profiling in the near future.

## Introduction

The increasing threat of tuberculosis (TB) drug resistance highlights the importance of prompt drug susceptibility test (DST) results for better patient care and infection control [1, 2]. Nevertheless, culture-dependent methods cannot provide a quick answer due to the fastidious nature of *Mycobacterium tuberculosis* (MTB). From literature, 24–61% of pulmonary TB cases were acid-fast bacilli (AFB) smear-negative [3, 4], with smear-negative, culture-positive TB accounting for 13% of TB transmission [5]. Novel diagnostic tools are needed for rapid detection of drug resistance from the technically demanding smear-negative specimens.

**Fig. 1:**
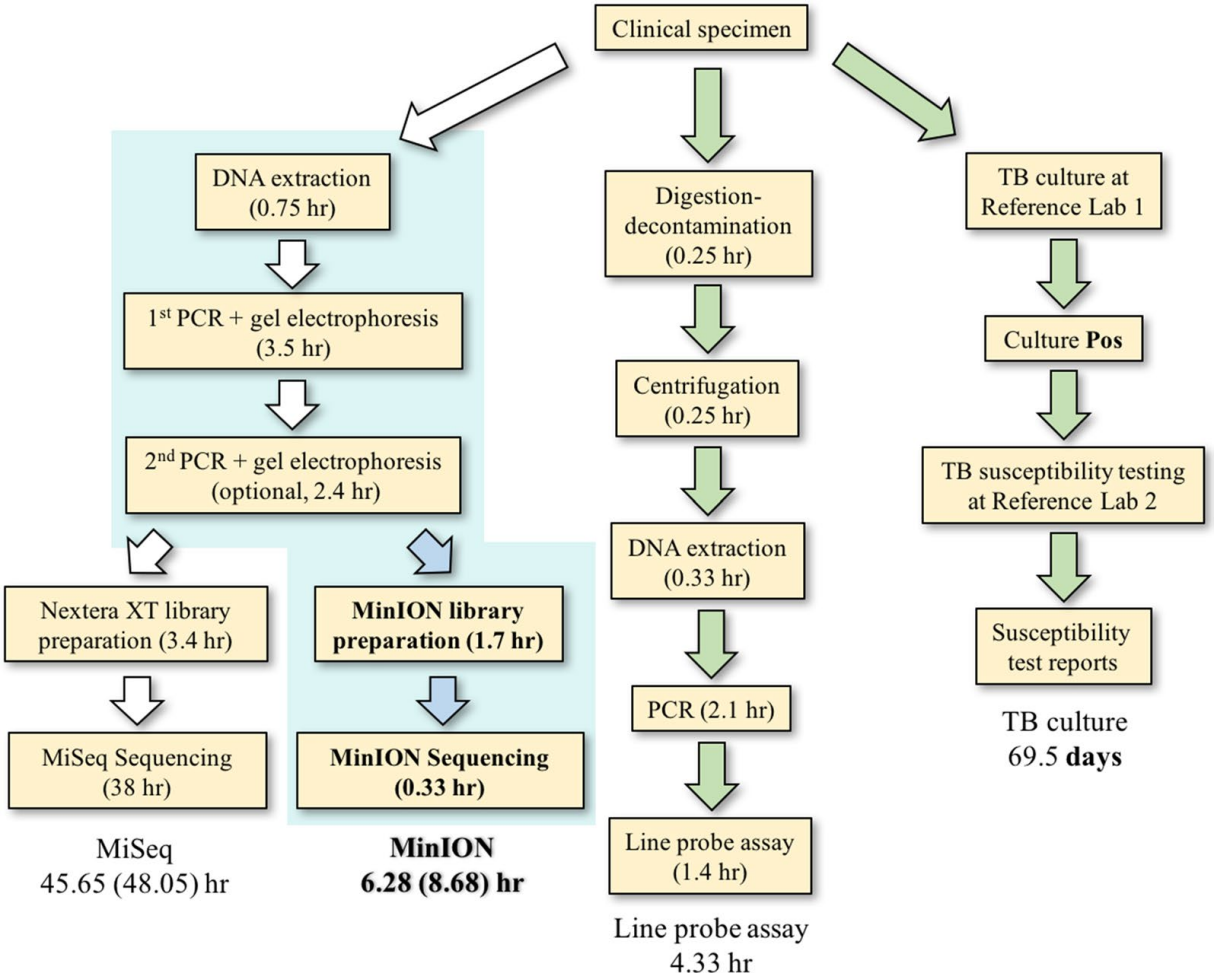
Assay time comparison between MiSeq, MinION, line probe assay and culture-dependent workflows

Recent advent of next-generation sequencing (NGS) has facilitated comprehensive evaluation of MTB genome for drug resistance prediction [6]. Among various options in NGS market, Nanopore sequencers are ideal for infectious disease diagnosis, which requires short sample-to-answer time (Fig. 1). Despite its inferior sequencing accuracy [7], several groups utilized Nanopore MinION and successfully identified single nucleotide variants (SNVs) in *Plasmodium falciparum* [8], dengue virus [9] and chronic lymphocytic leukemia [10, 11].

The goal of this study was to design and test a Nanopore targeted panel for direct TB drug resistance profiling. First, we attempted to amplify 10 resistance-associated loci from clinical specimens with low MTB burden. Second, we sequenced these amplicons with both Nanopore MinION and Illumina MiSeq, and the sequencing data was collated.

## Main text

### Methods

#### Clinical specimens

Twenty-three specimens were collected between August 2016 and May 2017 (Table 1). They were AFB smear-negative or classified as MTB detected low/very low by Xpert MTB/RIF assay (Cepheid, Sunnyvale, CA, USA). TB culture and DST were performed by 2 local reference laboratories, with ethambutol, isoniazid, rifampicin and streptomycin for first-line drug testing.

**Table 1:** Details of clinical specimens, routine test results and amplification of genomic regions associated with TB drug resistance

Patient	Specimen	Residual quantity	AFB smear	Xpert MTB/RIF results		Culture results^a^	Drug resistance panel (10 loci)^b^	
				MTB load	RIF		Amplified loci	Total number
1	BA	1.8 mL	Neg	Low	ND	All susceptible	All amplified	10
2	Lung Bx	4 pieces (~ 1 mm^3^)	Neg	Very low	Ind	All susceptible	*gyrA, rpoB, rpsL* & *tlyA*^c^	4
3	Sputum	~ 0.1 mL	Neg	Very low	ND	All susceptible	All amplified	10
4	BA	4.05 mL	Neg	Very low	ND	No growth	All amplified^d^	10
5	Sputum	0.35 mL	N/A	Very low	ND	N/A	*gyrA*	1
6	Sputum	0.2 mL	N/A	Very low	ND	N/A	*rpsL*	1
7	Bone marrow	~ 0.1 mL	N/A	Very low	ND	N/A	Nil	0
8	BA	3 mL	Neg	Low	ND	All susceptible	All amplified	10
9	BA	4.25 mL	Neg	Low	ND	All susceptible	All amplified	10
10	LN FNA	1.9 mL	Neg	Very low	ND	SM-resistant	*inhA*^P^ & *tlyA*^c^	2
11	LN FNA	1.5 mL	Neg	Low	ND	No growth	*eis*^P^*, inhA*^P^*, gyrA, katG, rpoB, rpsL, rrs* & *tlyA*^c^	8
12	BA	3 mL	Neg	Low	ND	No growth	All amplified	10
13	BA	4.5 mL	Neg	Low	ND	All susceptible	All amplified	10
14	Sputum	3 mL	Neg	Low	ND	SM-resistant	All amplified	10
15	LN FNA	1.6 mL	Neg	Low	ND	All susceptible	All amplified	10
16	LN FNA	1.5 mL	Neg	Low	ND	No growth	All amplified	10
17	BA	1 mL	Neg	Very low	ND	All susceptible	*embB*	1
18	Sputum	~ 0.1 mL	Neg	N/A		INH- & SM-resistant	*embB*	1
19	Lung Bx	0.2 mL	Pos	Low	ND	INH-resistant	All amplified	10
20	Cervical LN Bx	1 piece (~ 1 mm^3^)	Neg	N/A		All susceptible	Nil	0
21	BA	1.5 mL	Neg	Low	ND	All susceptible	All amplified	10
22	Cervical LN Bx	0.1 mL	Neg	Very low	ND	All susceptible	*eis*^P^*, inhA*^P^*, embB, gyrA, katG, rpoB, rpsL, rrs* & *tlyA*	9
23	BA	1.3 mL	Neg	N/A		All susceptible	*embB*	1
						Mean TAT: 69.5 days	All amplified	12 cases

*AFB* acid-fast bacilli, *BA* bronchial aspirate, *Bx* biopsy, *eis*^P^ *eis* promoter, *FNA* fine needle aspirate, *Ind* indeterminate, *INH* isoniazid, *inhA*^P^: *inhA* promoter, *LN* lymph node, *MTB M. tuberculosis, N/A* not available, *ND* not detected, *Neg* negative, *Pos* positive, *RIF* rifampicin resistance, *SM* streptomycin, *TAT* turnaround time

^a^Four first-line antibiotics were tested by local reference laboratory, including ethambutol, isoniazid, rifampicin and streptomycin

^b^The 10 loci were *eis* promoter*, embB, gyrA, inhA* promoter, *katG, pncA, rpoB, rpsL, rrs* and *tlyA* genes

^c^Negative by first PCR, positive by second PCR

^d^*eis* promoter and *rrs*: negative by first PCR, positive by second PCR

#### DNA extraction

Standard laboratory practice was applied to minimize the risk of infection and contamination. NucliSENS easyMAG automated system (bioMérieux, Marcy, I’Etoile, France) was used for DNA extraction. A maximum of 1-mL pretreated specimen was homogenized in lysis buffer and incubated at 80 °C for 20 min. DNA extraction was performed according to manufacturer’s recommendations, with an elution volume of 25 μL.

#### Amplification of resistance-associated loci

The 10 targets were located in *embB*, *gyrA*, *katG*, *pncA*, *rpoB*, *rpsL*, *rrs, tlyA* and promoter regions of *eis* and *inhA* genes. They were amplified in separate 25-μL polymerase chain reactions (PCRs), each comprising 1X PCR Buffer II, 1.5 mM MgCl_2_, 0.2 mM dNTPs, 1 μM forward and reverse primers (Additional file 1: Table S1), 2.5 U AmpliTaq Gold DNA polymerase (Applied Biosystems, Foster City, CA, USA), 1 M betaine (Sigma-Aldrich, St. Louis, MO, USA) and 2 μL of DNA. PCR conditions were as shown in Additional file 1: Table S2. Second PCR was performed if first PCR failed to yield the desired amplicon.

#### Sequencing by MiSeq

For each specimen, 2-μL aliquots of each amplicon were pooled for Nextera XT DNA library preparation (Illumina, San Diego, CA, USA). Indexed libraries were sequenced using MiSeq v3 Reagent Kit (Illumina, San Diego, CA, USA) in a 250–200 paired-end run.

#### Sequencing by MinION

Sequencing libraries were prepared using Ligation Sequencing Kit 1D R9 Version (Oxford Nanopore Technologies, Oxford, England). Purified libraries were sequenced on FLO-MIN 106 R9 flow cells (Oxford Nanopore Technologies, Oxford, England).

#### MiSeq data analysis

Sequencing reads were quality-filtered using Trimmomatic (Galaxy version 0.36.0) [12] and mapped to MTB reference sequence (NC_000962.3) using Burrows-Wheeler Aligner MEM algorithm (UGENE version 1.29.0) [13]. Samtools mpileup (Galaxy version 2.1.4) was used to generate pileups for BAM files [14]. SNVs, insertions and deletions (indels) were detected using VarScan mpileup (Galaxy version 2.4.3.1) with minimum coverage setting of 20, or by manual inspection using Integrative Genomics Viewer (IGV) version 2.4.13 with coverage allele-fraction threshold of 0.1 [15–17]. Sequencing coverage was estimated using Tablet version 1.17.08.17 [18]. SNVs and indels were correlated with DST results if available.

#### MinION data analysis

MinKNOW version 2.0 was used for live basecalling. Adaptor sequences were removed using Porechop (Galaxy version 0.2.3). Trimmed reads were mapped to MTB reference sequence (NC_000962.3) using minimap2 (Galaxy version 2.12) [19]. Sequencing error rate was estimated using Qualimap version 2.2.1 [20]. IGV was used for manual inspection of BAM datasets. Nanopolish variants (Galaxy version 0.1.0) was used for signal-level variant calling [21].

### Results and discussion

#### Routine test results

Nineteen specimens were AFB smear-negative, and the only smear-positive specimen was ‘MTB detected low’ (Patient 19) (Table 1). Twenty specimens were tested with Xpert MTB/RIF assay, with mean threshold cycle (Ct) values of 23.74–34.68, and rifampicin resistance was not detected in 19 but indeterminate for a lung biopsy with the lowest mean Ct value (Patient 2). TB culture was performed for 20 specimens, 16 were positive. Twelve of these isolates were susceptible to all first-line drugs tested, with isoniazid and streptomycin resistance detected in 2 and 3 isolates, respectively. Mean turnaround time of DST was 69.5 days.

#### Amplification of genomic targets

The targets were amplified in 64.35% of reactions (148/230) (Table 1). Success rate was the highest for *embB, gyrA, rpsL* and *tlyA* genes (16/23, 69.57%), while *pncA* gene was the lowest (12/23, 52.17%). At least 8 loci were successfully amplified from 14 specimens for predicting first- and second-line drug resistance (14/23, 60.87%), which was comparable to an 8-gene pyrosequencing assay for smear-negative specimens (54.9%) [22]. Twelve of these specimens (12/23, 52.17%) had all 10 loci amplified, with mean Ct values of 23.74–30.08. For the 8 specimens that did not yield all targets, the mean Ct values ranged from 28.16 to 34.68. It appeared that a Ct value of 28 or less was required for successful amplification of all targets.

#### MiSeq sequencing results

A total of 11,350,165 reads (4623 Mb) were generated, averaging 945,847 reads (385.3 Mb) per sample (Additional file 1: Table S3). Mean coverage breadth and depth per sample were 99.85% and 21,441.9, respectively. Minimum depth across all targets was 1488.

A total of 63 SNVs were called, 18 were synonymous, 25 were polymorphic, and 17 with unknown significance to drug resistance (Additional file 1: Table S4). The remaining 3 were missense mutations associated with quinolone, streptomycin and isoniazid resistance (Specimen 14, 16 and 19). No indels were called.

The MTB isolate from Specimen 14 was resistant to streptomycin, with wildtype *rpsL* and *rrs* genes. From literature, 25–52% of streptomycin-resistant MTB isolates harbored wildtype *rpsL* and *rrs* genes [23–25]. Other molecular mechanisms, such as reduced cell permeability to aminoglycosides or presence of drug-modifying enzymes, might contribute to its streptomycin resistance. On the other hand, 281A > G was called in *gyrA* gene with variant allelic frequency (VAF) of 11%. The resulting D94G substitution is frequently found in fluoroquinolone-resistant strains [26], yet we could not confirm the resistance phenotype as second-line drug susceptibility data was not available.

For Specimen 16, 128A > G was called in *rpsL* gene. We did not have any streptomycin susceptibility data for interpretation as TB culture was not performed.

For Specimen 19, − 15C > T was called in *inhA* promoter region, which is prevalent among isoniazid-resistant MTB isolates [27].

### MinION sequencing results

#### General features

Twelve pools of amplicons were sequenced separately on flow cells with 84–624 active pores (Additional file 1: Table S3). First FASTQ files were generated with an elapsed time of 5–86 min, 19.8 min in average, yielding 5.2–5.8 Mb of data and 2866–4685 ‘pass’ reads. Mean coverage breadth and depth across all targets were 100% and 282.8, respectively.

#### Sequencing error analysis

General alignment error rate was 10.47–15.12%, and mean insertion and deletion error rate (per 100 aligned bases) were 2.09% and 2.83%, respectively. The raw data error rate was comparable to previous studies for chronic lymphocytic leukemia [10, 11].

#### Comparison with MiSeq sequencing data

Nanopolish was used to improve the accuracy of MinION sequencing data from signal level. For *rpsL* amplicons of Patient 3 and 21, second and third FASTQ files were also included for data analysis to meet the default minimum depth requirement.

A total of 67,082 ‘nanopolished’ bases across all MinION consensus sequences were compared with MiSeq data (Additional file 1: Table S4 and S5). For the 67,019 wildtype bases called by MiSeq, 66,935 (99.9%) were called as wildtype and 84 (0.1%) as SNVs by MinION. For the 41 SNVs with 100% VAF called by MiSeq, 39 (95.1%) were called by MinION. For the 22 mixed bases called by MiSeq, a SNV (VAF: 70%) was called by MinION, yet the remainder were not called (VAF: 10–37%). There were 32 and 44 insertions and deletions from ‘nanopolished’ data, respectively.

#### Characteristics of discordant SNVs

The details are summarized in Additional file 1: Table S6, with following observations:

1. All discordant SNVs were wildtype bases by MiSeq, with allelic frequency of at least 99%.
2. A-G and C-T substitutions were involved, with higher frequency for the latter (Additional file 1: Fig. S1).
3. From MinION raw data, the ‘dominant’ bases (VAF: 51–95%) were identical to that of MiSeq, but were not called by Nanopolish.
4. From ‘nanopolished’ data, most discordant SNVs were present in *eis* promoter (n = 49) and absent in *gyrA*, *inhA* promoter, *rrs* and *tlyA* sequences (Additional file 1: Fig. S2).
5. From ‘nanopolished’ data, most discordant SNVs (80/84, 95.24%) were called recurrently in different specimens, and in 16 sequence patterns (Additional file 1: Fig. S3).

#### Characteristics of indels

The results are summarized in Additional file 1: Fig. S2 and S3, with following observations:

1. Most insertions were present in *eis* promoter (n = 9), whereas none was found in *inhA* promoter and *rpoB* sequences.
2. Most deletions were present in *katG* (n = 15) and absent in *gyrA*, *inhA* promoter, *pncA* and *rpsL* sequences.
3. Most indels (insertions, 75%; deletions, 90.91%) were called recurrently in different specimens (insertions, n = 7; deletions, n = 6).

#### Filtering recurrent variants

From ‘nanopolished’ data, recurrent variants were called across different samples, which was also observed in other studies using Nanopolish [10], nanocorrect and Amplicon Long-read Error Correction (ALEC) python script [11] for data processing. In both studies, the authors filtered recurrent variants to improve sequencing accuracy. By applying this strategy, the concordance for wildtype bases increased from 99.9% (66,935/67,019) to 100%, and insertions and deletions were reduced from 32 to 8 and 44 to 4, respectively (Additional file 1: Table S5).

## Conclusions

We developed a Nanopore targeted panel for direct detection of TB drug resistance, with full-set sequence data retrieved from about half of the specimens with low MTB burden. The assay time was 6–9 h, which improved an average of 69.5 days by DST, and was comparable to commercial methods like line probe assay (Fig. 1). The method was merited by reasonable reagent cost (64 USD per sample for 24-plex workflow), which could be further lowered with the 96-barcode option and Flongle flow cells [28, 29]. With continuously improving sequencing chemistry, more sophisticated signal correction pipelines and the above-mentioned merits, we envision that Nanopore sequencing can be a viable option for ‘end TB’ in the near future.

### Limitations

1. An increased sample size may better estimate the PCR success rate.
2. We could not correlate the data with second-line DST, which was not routinely performed for non-multidrug-resistant MTB isolates.
3. As the MinION flow cells possessed suboptimal number of active pores, the sequencing time might be overestimated.
4. Nanopore sequencing accuracy might be improved with ‘1D^2^′ chemistry, at the expense of lower sequencing depth [30].
5. Nanopolish could not improve the detection of minor variants, which might be caused by high error rate of Nanopore raw data. Sequencing error might originate from inhomogeneous translocation speed of DNA, low signal-to-noise ratio and simultaneous passage of multiple nucleotides through nanopores [31]. Nevertheless, substitution-type miscalls might also be present in MiSeq data, which might arise from similar emission spectra of fluorophores, phasing and pre-phasing phenomena, and index PCR of library preparation [32, 33]. In addition, polymerase error accumulated in target amplification could affect the accuracy of variant calling by both MiSeq and MinION.
6. Therefore, we should be vigilant that the variants called by MiSeq could be false whereas the variants called by MinION could be true in some occasions. It is advisable to confirm the presence of these variants by repeating the target amplification step followed by Sanger sequencing.

## Supplementary information

**Additional file 1: Fig. S1** Types of discordant SNVs from ‘nanopolished’ consensus sequences. **Fig. S2** Number of discordant SNVs and indels per gene in ‘nanopolished’ consensus sequences. **Fig. S3** Recurrent variants in ‘nanopolished’ consensus sequences. The bases in square brackets denote the positions of SNVs and indels. **Table S1** Primers used in this study. **Table S2** PCR conditions. **Table S3** General features of MiSeq and MinION sequencing runs. **Table S4** Single nucleotide variants (SNVs) called by MiSeq and corresponding MinION results. **Table S5** Comparison of MiSeq and MinION data. **Table S6** Details of discordant SNVs by MinION.

## Data Availability

The MinION and MiSeq sequencing reads were deposited in NCBI Sequence Read Archive (SRA) under the BioProject accession number PRJNA648373.

## Abbreviations

AFB: Acid-fast bacilli
ALEC: Amplicon long-read error correction
Ct: Threshold cycle
DST: Drug susceptibility testing
IGV: Integrative genomics viewer
Indels: Insertions and deletions
MTB: *Mycobacterium tuberculosis*
NGS: Next-generation sequencing
PCR: Polymerase chain reaction
SNV: Single nucleotide variant
TB: Tuberculosis
VAF: Variant allelic frequency
