# Supplementary material for: Rapid and economical drug resistance profiling with Nanopore MinION for clinical specimens with low bacillary burden of Mycobacterium tuberculosis
Source: BMC Res Notes. 2020 Sep 18;13:444. doi: 10.1186/s13104-020-05287-9 (PMC7501614; doi:10.1186/s13104-020-05287-9)
Supplement: Supplementary file 1 — Additional file 1: Fig. S1 Types of discordant SNVs from ‘nanopolished’ consensus sequences. Fig. S2 Number of discordant SNVs and indels per gene in ‘nanopolished’ consensus sequences. Fig. S3 Recurrent variants in ‘nanopolished’ consensus sequences. The bases in square brackets denote the positions of SNVs and indels. Table S1 Primers used in this study. Table S2 PCR conditions. Table S3 General features of MiSeq and MinION sequencing runs. Table S4 Single nucleotide variants (SNVs) called by MiSeq and corresponding MinION results. Table S5 Comparison of MiSeq and MinION data. Table S6 Details of discordant SNVs by MinION. [file 13104_2020_5287_MOESM1_ESM.docx]

**
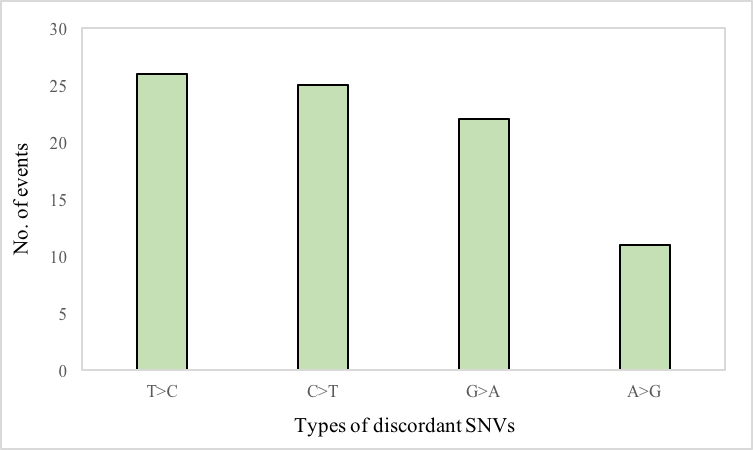
**

**Fig. S1** Types of discordant SNVs from ‘nanopolished’ consensus sequences.

**
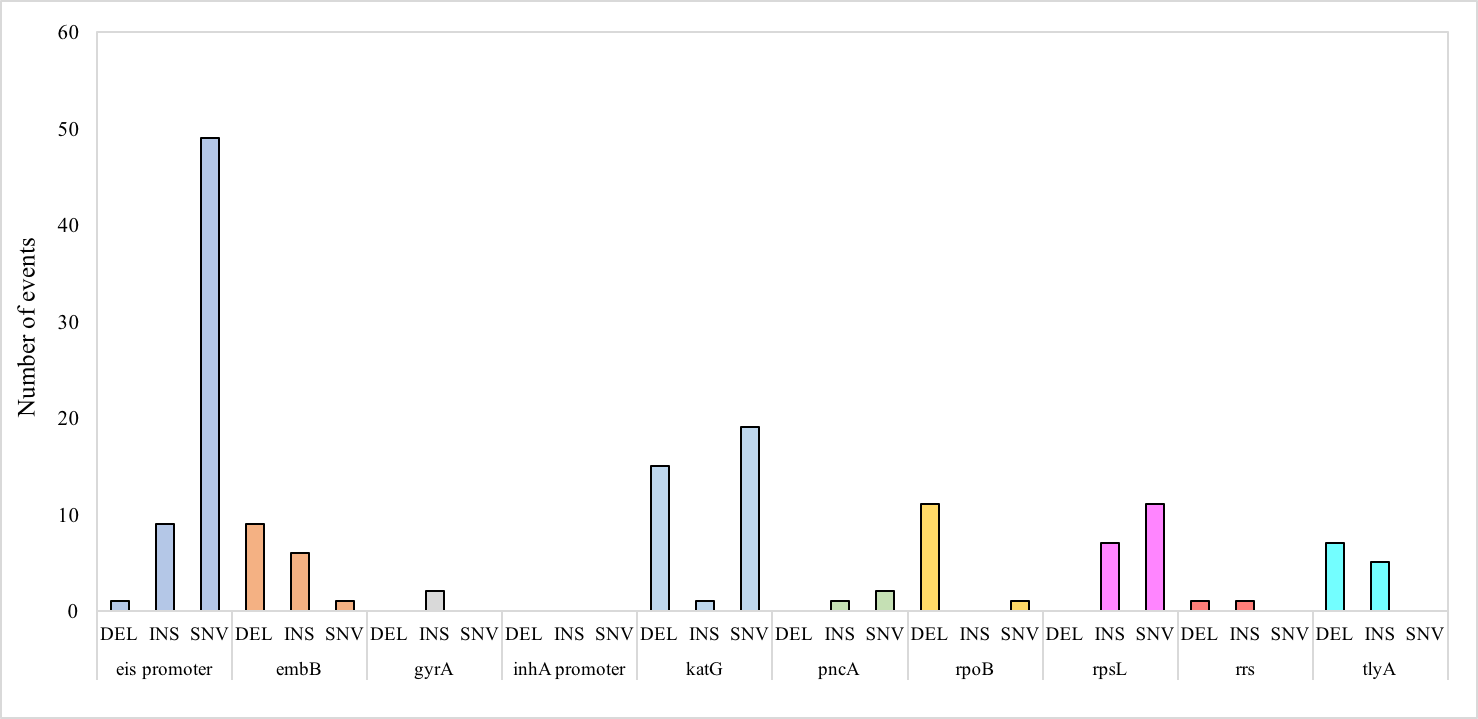
**

**Fig. S2** Number of discordant SNVs and indels per gene in ‘nanopolished’ consensus sequences.

**
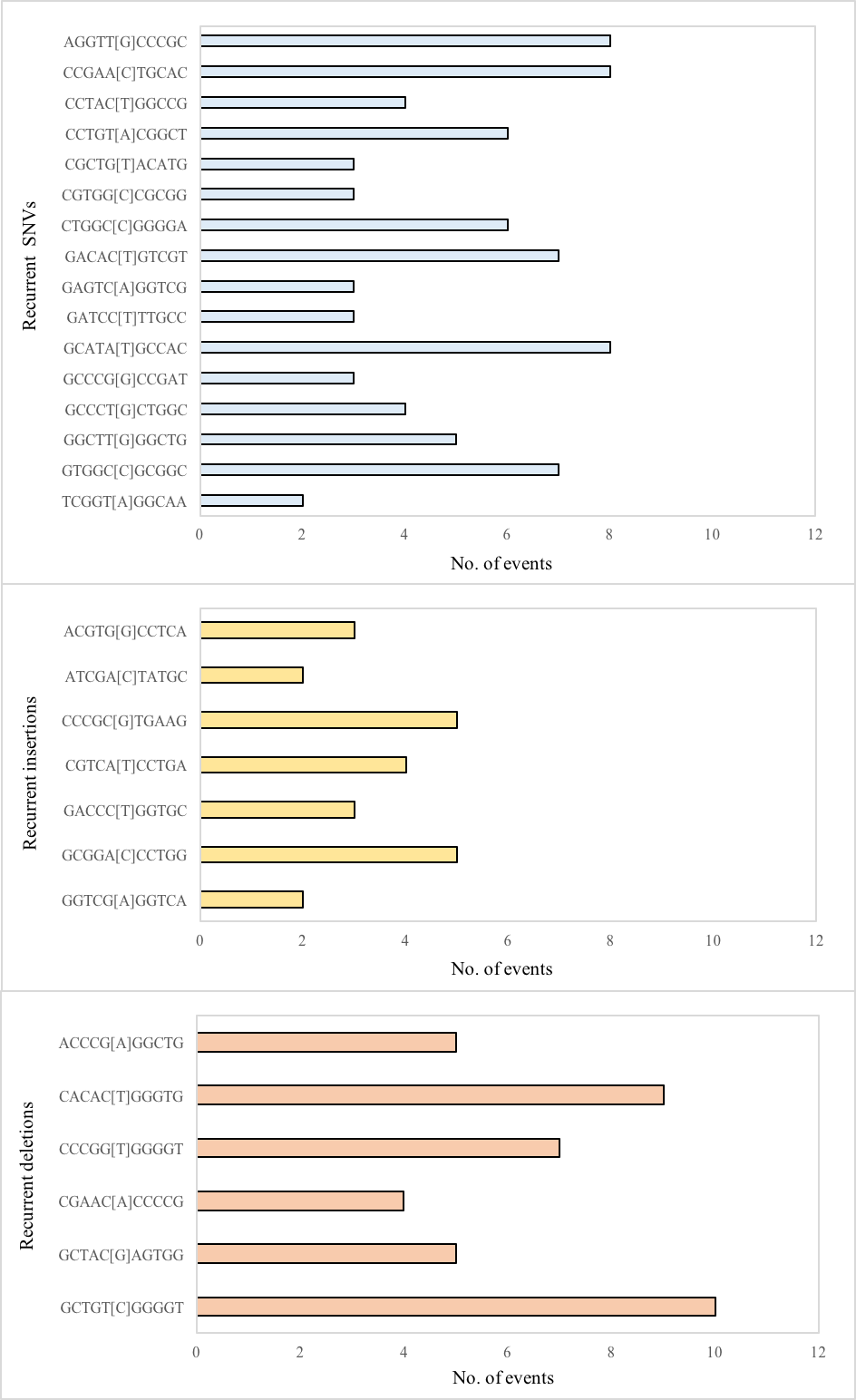
**

**Fig. S3** Recurrent variants in ‘nanopolished’ consensus sequences. The bases in square brackets denote the positions of SNVs and indels.

**Table S1** Primers used in this study.

| **Genes** | **PCR** | **Primer sequences (5’ to 3’)** | | **Amplicon size (bp)** | **References** |
| --- | --- | --- | --- | --- | --- |
|  |  | **Forward** | **Reverse** |  |  |
| ***eis***  **promoter** | 1^st^ | GCGTAACGTCACGGCGAAATTC | GTCAGCTCATGCAAGGTGGTAGC | 567 | [1] |
|  | 2^nd^ | Same as 1^st^ PCR | ATCCCGACCACCTCAGAACC | 327 | This study |
| ***embB*** | 1^st^ | CTGACCGACGCCGTGGTGATAT | CAGGATGAGGTAGTAGTAACGCAGG | 759 | Fp: [1]; Rp: this study |
|  | 2^nd^ | GGCTTCCTGCTCTGGCATGTC | Same as 1^st^ PCR | 735 | This study |
| ***gyrA*** | 1^st^ | CCCTGCGTTCGATTGCAAAC | CTTCGGTGTACCTCATCGCC | 423 | [1] |
|  | 2^nd^ | GACTCGCTCGACCGGATCG | TGGGTCATTGCCTGGCGAG | 342 | This study |
| ***inhA* promoter** | 1^st^ | CGAAGTGTGCTGAGTCACACCG | ATGAGGAATGCGTCCGCGGA | 430 | Fp: [2]; Rp: [1] |
|  | 2^nd^ | Same as 1^st^ PCR | GGACCCTGGTGCTCTTCTACC | 379 | This study |
| ***katG*** | 1^st^ | AACGACGTCGAAACAGCGGC | GCGAACTCGTCGGCCAATTC | 455 | [1] |
|  | 2^nd^ | GCTGATCGTCGGCGGTCAC | GGGTGTTCCAGCCAGCGAC | 411 | This study |
| ***pncA*** | 1^st^ | ACAGTTCATCCCGGTTCGGC | GGTCATGTTCGCGATCGTCG | 689 | [1] |
|  | 2^nd^ | GTGCCATCAGGAGCTGCAAAC | CGTCATGGACCCTATATCTGTGGC | 645 | This study |
| ***rpoB*** | 1^st^ | CTTGCACGAGGGTCAGACCA | ATCTCGTCGCTAACCACGCC | 543 | [1] |
|  | 2^nd^ | GTGGAAACCGACGACATCGACC | CGCCGTCGACCACCTTGC | 475 | This study |
| ***rpsL*** | 1^st^ | CAAGGGTCGTCGGGACAAGATC | TCTTGACACCCTGCGTATCCAGC | 299 | Modified from [3] |
|  | 2^nd^ | CGGCTCTGAAGGGCAGCC | GATGATCTTGTAGCGCACACCAGG | 230 | This study |
| ***rrs*** | 1^st^ | CCTTGTCTCATGTTGCCAGCACG | GTCCGAGTGTTGCCTCAGG | 570 | Fp: this study; Rp: [1] |
|  | 2^nd^ | GTCAACTCGGAGGAAGGTGG | GCTCTCGCCCACTACAGACAAG | 454 | Fp: [1]; Rp: this study |
| ***tlyA*** | 1^st^ | AGGCGCACGAGGTGTTGTTG | ACTTTTTCTACGCGCCGTGC | 946 | [1] |
|  | 2^nd^ | ATGTCGGATACGGCCAGCTG | Same as 1^st^ PCR | 555 | [1] |

Fp: forward primer; Rp: reverse primer; 1^st^: first; 2^nd^: second.

**Table S2** PCR conditions

| **First PCR** | | | |
| --- | --- | --- | --- |
| **Phase** | **Temperature** | **Time** | **Number of cycles** |
| Initial denaturation | 95 °C | 10 min | 1 |
| Phase 1  Touch-down PCR | 95 °C | 1 min | 20 |
|  | 70 °C to 62 °C (-0.4 °C/cycle) | 1 min |  |
|  | 72 °C | 45 s |  |
| Phase 2  Amplification | 95 °C | 1 min | 30 |
|  | 60 °C | 1 min |  |
|  | 72 °C | 45 s |  |
| Final extension | 72 °C | 5 min | 1 |
| Hold | 15 °C | ∞ | / |
| **Second PCR** | | | |
| **Phase** | **Temperature** | **Time** | **Number of cycles** |
| Initial denaturation | 95 °C | 10 min | 1 |
| Phase 1  Touch-down PCR | 95 °C | 30 s | 20 |
|  | 70 °C to 62 °C (-0.4 °C/cycle) | 30 s |  |
|  | 72 °C | 45 s |  |
| Phase 2  Amplification | 95 °C | 30 s | 20 |
|  | 60 °C | 30 s |  |
|  | 72 °C | 45 s |  |
| Final extension | 72 °C | 5 min | 1 |
| Hold | 15 °C | ∞ | / |

**Table S3** General features of MiSeq and MinION sequencing runs

| **Patient** | **MiSeq** | | | | **MinION** | | | | | |
| --- | --- | --- | --- | --- | --- | --- | --- | --- | --- | --- |
|  | **No. of reads** | **Yield (Mb)** | **Mean BOC** | **Mean depth** | **Active pores** | **Sequencing time (min)** | **‘Pass’ reads** | **Yield (Mb)** | **Mean BOC** | **Mean depth** |
| 1 | 1,281,762 | 460.6 | 99.93% | 26,575.7 | 224 | 8 | 4,478 | 5.2 | 100% | 361.2 |
| 3 | 1,512,122 | 535.7 | 99.95% | 33,896 | 147 | 86 | 3,164 | 5.3 | 100% | 221 |
| 4 | 432,428 | 172.1 | 99.61% | 10,080 | 461 | 10 | 4,229 | 5.3 | 100% | 310.4 |
| 8 | 1,084,109 | 394.8 | 100% | 24,496.7 | 161 | 5 | 4,299 | 5.5 | 100% | 241.7 |
| 9 | 925,106 | 372.6 | 100% | 19,397.2 | 360 | 27 | 4,083 | 5.2 | 100% | 281 |
| 12 | 927,446 | 360.9 | 99.98% | 22,473.3 | 384 | 10 | 3,726 | 5.3 | 100% | 283.8 |
| 13 | 910,791 | 540.8 | 100% | 21,089.6 | 397 | 21 | 2,866 | 5.4 | 100% | 208.9 |
| 14 | 997,515 | 377.3 | 99.99% | 24,635.2 | 230 | 21 | 3,785 | 5.3 | 100% | 291 |
| 15 | 356,841 | 363.6 | 99.97% | 8,029 | 368 | 9 | 2,979 | 5.3 | 100% | 219.2 |
| 16 | 1,090,271 | 380.9 | 99.99% | 26,859.3 | 624 | 9 | 4,685 | 5.3 | 100% | 337.6 |
| 19 | 614,571 | 246.2 | 98.75% | 13,503.2 | 435 | 23 | 4,069 | 5.4 | 100% | 326.9 |
| 21 | 1,217,203 | 417.9 | 100% | 26,267.9 | 84 | 8 | 4,370 | 5.8 | 100% | 310.8 |
| **Mean** | **945,847** | **385.3** | **99.85%** | **21,441.9** | **322.9** | **19.8** | **3,894.4** | **5.4** | **100%** | **282.8** |

BOC: breadth of coverage.

**Table S4** Single nucleotide variants (SNVs) called by MiSeq and corresponding MinION results

| **Patient** | **Gene** | **Nucleotide position** | **SNV** | **Coverage depth** | **Allelic frequency** | **Amino acid change?** | **MinION results** | **Remarks** |
| --- | --- | --- | --- | --- | --- | --- | --- | --- |
| 1 | *gyrA* | 61 | GAG/CAG | 3,544 | 100% | Codon 21: Glu/Gln | Detected | Polymorphism [4] |
|  |  | 284 | AGC/ACC | 8,675 | 100% | Codon 95: Ser/Thr | Detected |  |
|  | *tlyA* | 33 | CTA/CTG | 51,382 | 100% | Codon 11: Leu/Leu | Detected | Silent mutation |
| 3 | *embB* | 1180 | GCG/ACG | 20,326 | 37% | Codon 394: Ala/Thr | Not detected | Resistance pattern not documented |
|  | *gyrA* | 61 | GAG/CAG | 10,814 | 100% | Codon 21: Glu/Gln | Detected | Polymorphism [4] |
|  |  | 189 | GGC/GGT | 22,982 | 26% | Codon 63: Gly/Gly | Not detected | Silent mutation |
|  |  | 284 | AGC/ACC | 19,779 | 100% | Codon 95: Ser/Thr | Detected | Polymorphism [4] |
|  | *pncA* | 419 | CGC/CAC | 113,057 | 24% | Codon 140: Arg/His | Not detected | Polymorphism [5] |
|  | *rpoB* | 1150^a^ | CGG/TGG | 49,648 | 100% | Codon 465^b^: Arg/Trp | Detected | Resistance pattern not documented |
|  |  | 1424^a^ | GGC/GAC | 59,981 | 26% | Codon 556^b^: Gly/Asp | Not detected |  |
|  | *tlyA* | 33 | CTA/CTG | 17,465 | 100% | Codon 11: Leu/Leu | Detected | Silent mutation |
|  |  | 722 | CTG/CCG | 36,373 | 14% | Codon 241: Leu/Pro | Not detected | Resistance pattern not documented |
| 4 | *eis* | 143 | GTC/GAC | 3,680 | 13% | Codon 48: Val/Asp | Not detected | Resistance pattern not documented |
|  | *embB* | 345 | CTG/CTA | 9,560 | 11% | Codon 115: Leu/Leu | Not detected | Silent mutation |
|  | *gyrA* | 61 | GAG/CAG | 3,091 | 100% | Codon 21: Glu/Gln | Detected | Polymorphism [4] |
|  |  | 284 | AGC/ACC | 6,790 | 100% | Codon 95: Ser/Thr | Detected |  |
|  | *inhA* promoter | -101 | C/T | 5,920 | 10% | / | Not detected | Resistance pattern not documented |
|  | *fabG1* | 216 | TTC/TTT | 8,126 | 15% | Codon 72: Phe/Phe | Not detected | Silent mutation |
|  |  | 232 & 234 | CAC/TAT | 6,514 & 6,473 | 16% | Codon 78: His/Tyr | Not detected | Resistance pattern not documented |
|  |  | 271 | CTA/TTA | 1,781 | 19% | Codon 91: Leu/Leu | Not detected | Silent mutation |
|  | *rpsL* | 186 | GAG/GAA | 3,428 | 14% | Codon 62: Glu/Glu | Not detected | Silent mutation |
|  | *tlyA* | 33 | CTA/CTG | 1,399 | 100% | Codon 11: Leu/Leu | Detected | Silent mutation |
| 8 | *eis* promoter | -50 | G/A | 18,178 | 14% | / | Not detected | Resistance pattern not documented |
|  | *eis* | 95 | TCA/TTA | 45,814 | 24% | Codon 32: Ser/Leu | Not detected | Resistance pattern not documented |
|  | *gyrA* | 61 | GAG/CAG | 6,242 | 100% | Codon 21: Glu/Gln | Detected | Polymorphism [4] |
|  |  | 284 | AGC/ACC | 13,040 | 100% | Codon 95: Ser/Thr | Detected |  |
|  | *rpoB* | 1,055 | CCG/CTG | 13,777 | 100% | Codon 433: Pro/Leu | Not detected | Resistance pattern not documented |
|  | *tlyA* | 33 | CTA/CTG | 10,876 | 100% | Codon 11: Leu/Leu | Detected | Silent mutation |
| 9 | *eis* | 309 | GAA/GAC | 12,526 | 100% | Codon 103: Glu/Asp | Not detected | Resistance pattern not documented |
|  | *gyrA* | 61 | GAG/CAG | 5,276 | 100% | Codon 21: Glu/Gln | Detected | Polymorphism [4] |
|  |  | 284 | AGC/ACC | 12,879 | 100% | Codon 95: Ser/Thr | Detected |  |
|  | *tlyA* | 33 | CTA/CTG | 27,066 | 100% | Codon 11: Leu/Leu | Detected | Silent mutation |
|  |  | 232 | GCG/ACG | 53,574 | 13% | Codon 78: Ala/Thr | Not detected | Resistance pattern not documented |
|  |  | 691 | CCG/TCG | 53,778 | 12% | Codon 231: Pro/Ser | Not detected |  |
| 12 | *gyrA* | 61 | GAG/CAG | 7,232 | 100% | Codon 21: Glu/Gln | Detected | Polymorphism [4] |
|  |  | 284 | AGC/ACC | 15,836 | 100% | Codon 95: Ser/Thr | Detected |  |
|  | *pncA* | 106 | GCC/ACC | 28,528 | 20% | Codon 36: Ala/Thr | Not detected | Resistance pattern not documented |
|  | *tlyA* | 33 | CTA/CTG | 3,123 | 100% | Codon 11: Leu/Leu | Detected | Silent mutation |
| 13 | *gyrA* | 61 | GAG/CAG | 7,031 | 100% | Codon 21: Glu/Gln | Detected | Polymorphism [4] |
|  |  | 284 | AGC/ACC | 15,255 | 100% | Codon 95: Ser/Thr | Detected |  |
|  | *tlyA* | 33 | CTA/CTG | 13,592 | 100% | Codon 11: Leu/Leu | Detected | Silent mutation |
| 14 | *gyrA* | 61 | GAG/CAG | 7,237 | 100% | Codon 21: Glu/Gln | Detected | Polymorphism [4] |
|  |  | 281 | GAC/GGC | 18,655 | 11% | Codon 94: Asp/Gly | Not detected | Elevated CIP MIC [6] |
|  |  | 284 | AGC/ACC | 17,899 | 100% | Codon 95: Ser/Thr | Detected | Polymorphism [4] |
|  | *tlyA* | 33 | CTA/CTG | 8,160 | 100% | Codon 11: Leu/Leu | Detected | Silent mutation |
| 15 | *gyrA* | 61 | GAG/CAG | 3,170 | 100% | Codon 21: Glu/Gln | Detected | Polymorphism [4] |
|  |  | 284 | AGC/ACC | 7,235 | 100% | Codon 95: Ser/Thr | Detected |  |
|  | *inhA* promoter | -67 | G/A | 10,278 | 11% | / | Not detected | Resistance pattern not documented |
|  |  | -21 | G/T | 12,501 | 12% | / | Not detected |  |
|  | *fabG1* | 219 | ACC/ACT | 5,802 | 18% | Codon 73: Thr/Thr | Not detected | Silent mutation |
|  | *tlyA* | 33 | CTA/CTG | 2,492 | 100% | Codon 11: Leu/Leu | Detected | Silent mutation |
|  |  | 311 | GGT/GAT | 6,094 | 70% | Codon 104: Gly/Asp | Detected | Resistance pattern not documented |
| 16 | *gyrA* | 61 | GAG/CAG | 6,564 | 100% | Codon 21: Glu/Gln | Detected | Polymorphism [4] |
|  |  | 284 | AGC/ACC | 16,601 | 100% | Codon 95: Ser/Thr | Detected |  |
|  | *rpsL* | 128 | AAG/AGG | 40,431 | 100% | Codon 43: Lys/Arg | Detected | SM resistance [7] |
|  | *tlyA* | 33 | CTA/CTG | 12,168 | 100% | Codon 11: Leu/Leu | Detected | Silent mutation |
| 19 | *gyrA* | 61 | GAG/CAG | 6,276 | 100% | Codon 21: Glu/Gln | Detected | Polymorphism [4] |
|  |  | 284 | AGC/ACC | 10,377 | 100% | Codon 95: Ser/Thr | Detected |  |
|  | *inhA* promoter | -15 | C/T | 12,946 | 100% | / | Detected | INH resistance [8] |
|  | *tlyA* | 33 | CTA/CTG | 11,787 | 100% | Codon 11: Leu/Leu | Detected | Silent mutation |
| 21 | *gyrA* | 61 | GAG/CAG | 9,131 | 100% | Codon 21: Glu/Gln | Detected | Polymorphism [4] |
|  |  | 284 | AGC/ACC | 20,326 | 100% | Codon 95: Ser/Thr | Detected |  |
|  | *tlyA* | 33 | CTA/CTG | 21,007 | 100% | Codon 11: Leu/Leu | Detected | Silent mutation |

^a^ Nucleotide position on *M. tuberculosis rpoB* gene.

^b^ Codon position relative to *E. coli rpoB* sequence.

CIP, ciprofloxacin; MIC, minimum inhibitory concentration.

**Table S5** Comparison of MiSeq and MinION data

| **SNVs** | | | | | | |
| --- | --- | --- | --- | --- | --- | --- |
| **Genotypes** | **MiSeq results** | | **MinION results** | | | |
|  |  |  | **Nanopolish only** | | **Nanopolish + filtering recurrent variants** | |
|  | **VAF** | **No. of nucleotides** | **Matched** | **Not matched** | **Matched** | **Not matched** |
| Wildtype | / | 67,019 | 66,935 (99.9%) | 84 (0.1%) | 67,019 (100%) | 0 |
| Variant | 100% | 41 | 39 (95.1%) | 2 (4.9%) | 39 (95.1%) | 2 (4.9%) |
| Mixed | > 20%, < 70% | 7 | 1 (14.3%) | 6 (85.7%) | 1 (14.3%) | 6 (85.7%) |
| Mixed | > 10%, < 20% | 15 | 0 | 15 (100%) | 0 (0%) | 15 (100%) |
| **Insertions** | | | | | | |
|  | **MiSeq results** | | **MinION results** | | | |
|  |  |  | **Nanopolish only** | | **Nanopolish + filtering recurrent variants** | |
| No. of insertions | 0 | | 32 | | 8 | |
| **Deletions** | | | | | | |
|  | **MiSeq results** | | **MinION results** | | | |
|  |  |  | **Nanopolish only** | | **Nanopolish + filtering recurrent variants** | |
| No. of deletions | 0 | | 44 | | 4 | |

VAF, variant allelic frequency

**Table S6** Details of discordant SNVs by MinION

| **Patient** | **Gene** | **Nucleotide position** | **VAF** | | |
| --- | --- | --- | --- | --- | --- |
|  |  |  | **Nanopolish** | **MinION raw data** | **MiSeq** |
| 1 | *eis* promoter | -55 | T: 37%; C: 58.3% | T: 66%; C: 33% | T: 100% |
|  |  | -27 | C: 38.6%; T: 58.4% | C: 82%; T: 16% | C: 100% |
|  |  | -17 | T: 35.4%; C: 52.9% | T: 84%; C: 14% | T: 100% |
|  |  | 56 | T: 44.3%; C: 50.8% | T: 63%; C: 35% | T: 100% |
|  |  | 190 | T: 38.8%; C: 56.5% | T: 83%; C: 12% | T: 100% |
|  | *katG* | 841 | G: 39.4%; A: 52.7% | G: 92%; A: 6% | G: 100% |
|  |  | 1,010 | A: 25.5%; G: 75.5% | A: 81%; G: 18% | A: 100% |
|  | *rpsL* | 179 | A: 29%; G: 63.1% | A: 87%; G: 13% | A: 100% |
| 3 | *eis* promoter | -27 | C: 33.6%; T: 58.7% | C: 68%; T: 27% | C: 100% |
|  |  | 310 | C: 40.2%; T: 56.6% | C: 76%; T: 23% | C: 100% |
|  | *katG* | 841 | G: 37.4%; A: 55.1% | G: 90%; A: 6% | G: 100% |
|  |  | 1,010 | A: 39.4%; G: 59.1% | A: 79%; G: 20% | A: 100% |
| 4 | *eis* promoter | -26 | C: 33.6%; T: 62.6% | C: 68%; T: 21% | C: 100% |
|  |  | 41 | C: 46.5%; T: 50.8% | C: 94%; T: 6% | C: 100% |
|  | *katG* | 894 | G: 35.4%; A: 58.2% | G: 56%; A: 42% | G: 100% |
|  | *rpsL* | 157 | G: 34%; A: 64.6% | G: 93%; A: 5% | G: 100% |
| 8 | *eis* promoter | -67 | T: 45.4%; C: 52.5% | T: 85%; C: 10% | T: 100% |
|  |  | 190 | T: 43.7%; C: 50.8% | T: 67%; C: 25% | T: 100% |
|  | *katG* | 841 | G: 28%; A: 65% | G: 87%; A: 9% | G: 100% |
|  |  | 854 | G: 37.2%; A: 53.4% | G: 91%; A: 9% | G: 100% |
|  |  | 1,010 | A: 34.5%; G: 63.9% | A: 79%; G: 19% | A: 100% |
|  | *pncA* | 397 | A: 38.3%; G: 56.1% | A: 71%; G: 26% | A: 100% |
|  | *rpoB* | 1,633 | C: 36.7%; T: 60.2% | C: 91%; T: 8% | C: 100% |
|  | *rpsL* | 157 | G: 37.6%; A: 56.8% | G: 89%; A: 11% | G: 100% |
| 9 | *eis* promoter | -55 | T: 42%; C: 55.7% | T: 66%; C: 33% | T: 100% |
|  |  | -26 | C: 40.8%; T: 56.8% | C: 76%; T: 17% | C: 100% |
|  |  | -17 | T: 44.1%; C: 46.8% | T: 63%; C: 32% | T: 100% |
|  |  | 41 | C: 41.1%; T: 56.5% | C: 95%; T: 5% | C: 100% |
|  |  | 310 | C: 6.5%; T: 83.2% | C: 94%; T: 5% | C: 100% |
|  | *katG* | 894 | G: 33.8%; A: 62.2% | G: 51%; A: 48% | G: 100% |
|  | *rpsL* | 157 | G: 33.8%; A: 64.1% | G: 86%; A: 14% | G: 100% |
| 12 | *eis* promoter | -67 | T: 35.8%; C: 63.3% | T: 89%; C: 7% | T: 100% |
|  |  | -55 | T: 31%; C: 66.9% | T: 58%; C: 42% | T: 100% |
|  |  | -26 | C: 34.6%; T: 61.4% | C: 66%; T: 26% | C: 100% |
|  |  | -17 | T: 29.7%; C: 62.8% | T: 71%; C: 27% | T: 99%; C: 1% |
|  |  | 41 | C: 35.8%; T: 61.1% | C: 93%; T: 6% | C: 100% |
|  |  | 310 | C: 35.6%; T: 62.3% | C: 88%; T: 12% | C: 100% |
|  | *katG* | 894 | G: 37.1%; A: 57.4% | G: 57%; A: 42% | G: 100% |
|  |  | 1,042 | G: 40.9%; A: 56.7% | G: 89%; A: 11% | G: 100% |
|  | *pncA* | 397 | A: 41%; G: 55.4% | A: 80%; G: 19% | A: 100% |
|  | *rpsL* | 157 | G: 36.9%; A: 61.5% | G: 77%; A: 22% | G: 100% |
|  |  | 179 | A: 37.8%; G: 59.4% | A: 86%; G: 14% | A: 100% |
| 13 | *eis* promoter | -55 | T: 36.3%; C: 61.2% | T: 61%; C: 39% | T: 100% |
|  |  | -26 | C: 39%; T: 52.9% | C: 76%; T: 17% | C: 100% |
|  |  | -17 | T: 39.7%; C: 50.4% | T: 82%; C: 16% | T: 100% |
|  |  | 41 | C: 43.4%; T: 52.4% | C: 94%; T: 5% | C: 100% |
|  |  | 56 | T: 42.3%; C: 53.5% | T: 60%; C: 39% | T: 100% |
|  |  | 310 | C: 39.7%; T: 56.4% | C: 87%; T: 13% | C: 100% |
|  | *embB* | 1,469 | T: 38.5%; C: 55.9% | T: 85%; C: 13% | T: 100% |
|  | *katG* | 894 | G: 41.3%; A: 44.8% | G: 72%; A: 26% | G: 100% |
|  |  | 1,010 | A: 39.2%; G: 59.6% | A: 84%; G: 14% | A: 100% |
|  |  | 1,042 | G: 43.2%; A: 54.2% | G: 88%; A: 11% | G: 100% |
|  | *rpsL* | 157 | G: 38.5%; A: 58% | G: 79%; A: 20% | G: 100% |
| 14 | *eis* promoter | -55 | T: 34.8%; C: 63.1% | T: 64%; C: 35% | T: 100% |
|  |  | -26 | C: 37.6%; T: 57.4% | C: 71%; T: 23% | C: 100% |
|  |  | -17 | T: 29.2%; C: 60.2% | T: 73%; C: 25% | T: 100% |
|  |  | 56 | T: 45%; C: 49.3% | T: 62%; C: 36% | T: 100% |
|  |  | 310 | C: 36.6%; T: 59.2% | C: 80%; T: 19% | C: 100% |
|  | *katG* | 1,010 | A: 37.6%; G: 60.5% | A: 86%; G: 13% | A: 100% |
|  | *rpsL* | 157 | G: 37.7%; A: 61.5% | G: 81%; A: 19% | G: 100% |
| 15 | *eis* promoter | -55 | T: 39.8%; C: 57.2% | T: 65%; C: 35% | T: 100% |
|  |  | -26 | C: 41.2%; T: 52.7% | C: 77%; T: 17% | C: 100% |
|  |  | -17 | T: 32.4%; C: 59% | T: 73%; C: 25% | T: 100% |
|  |  | 310 | C: 35.6%; T: 63.5% | C: 86%; T: 13% | C: 100% |
|  | *rpsL* | 157 | G: 38%; A: 60.2% | G: 75%; A: 25% | G: 100% |
| 16 | *eis* promoter | -67 | T: 42.8%; C: 55.4% | T: 83%; C: 11% | T: 100% |
|  |  | -55 | T: 34.5%; C: 61.2% | T: 51%; C: 49% | T: 100% |
|  |  | -27 | C: 38.9%; T: 55.7% | C: 77%; T: 21% | C: 100% |
|  |  | -17 | T: 33.2%; C: 57% | T: 83%; C: 14% | T: 100% |
|  |  | 41 | C: 40.1%; T: 52.6% | C: 94%; T: 6% | C: 100% |
|  |  | 56 | T: 35.5%; C: 59.5% | T: 55%; C: 43% | T: 100% |
|  |  | 190 | T: 39.8%; C: 55.7% | T: 83%; C: 15% | T: 100% |
|  |  | 310 | C: 40.3%; T: 55.3% | C: 83%; T: 15% | C: 100% |
|  | *katG* | 1,010 | A: 37.7%; G: 60.3% | A: 85%; G: 14% | A: 100% |
|  |  | 1,042 | G: 41.8%; A: 54.2% | G: 84%; A: 15% | G: 100% |
|  | *rpsL* | 84 | G: 38.8%; A: 54.8% | G: 58%; A: 38% | G: 100% |
|  |  | 179 | A: 22.9%; G: 70.4% | A: 69%; G: 30% | A: 100% |
| 19 | *eis* promoter | -26 | C: 41.8%; T: 56.2% | C: 74%; T: 20% | C: 100% |
|  |  | -17 | T: 27.5%; C: 67.1% | T: 74%; C: 24% | T: 100% |
|  |  | 310 | C: 27.7%; T: 68% | C: 83%; T: 17% | C: 100% |
|  | *katG* | 894 | G: 39%; A: 55.2% | G: 60%; A: 39% | G: 100% |
|  | *rpsL* | 157 | G: 28.7%; A: 69.6% | G: 82%; A: 17% | G: 100% |
| 21 | *eis* promoter | 41 | C: 39.8%; T: 53.3% | C: 93%; T: 6% | C: 100% |
|  | *katG* | 1042 | G: 35.8%; A: 61.2% | G: 87%; A: 12% | G: 100% |

**References:**

1. Campbell PJ, Morlock GP, Sikes RD, Dalton TL, Metchock B, Starks AM, et al. Molecular detection of mutations associated with first- and second-line drug resistance compared with conventional drug susceptibility testing of *Mycobacterium tuberculosis*. Antimicrob Agents Chemother. 2011; 55(5):2032-41.
2. Herrera-León L, Molina T, Saíz P, Sáez-Nieto JA, Jiménez MS. New multiplex PCR for rapid detection of isoniazid-resistant *Mycobacterium tuberculosis* clinical isolates. Antimicrob Agents Chemother. 2005; 49(1):144-7.
3. Zheng R, Zhu C, Guo Q, Qin L, Wang J, Lu J, et al. Pyrosequencing for rapid detection of tuberculosis resistance in clinical isolates and sputum samples from re-treatment pulmonary tuberculosis patients. BMC Infect Dis. 2014; 14:200.
4. Devasia R, Blackman A, Eden S, Li H, Maruri F, Shintani A, et al. High proportion of fluoroquinolone-resistant *Mycobacterium tuberculosis* isolates with novel gyrase polymorphisms and a *gyrA* region associated with fluoroquinolone susceptibility. J Clin Microbiol. 2012; 50(4):1390-6.
5. Ando H, Mitarai S, Kondo Y, Suetake T, Sekiguchi JI, Kato S, et al. Pyrazinamide resistance in multidrug-resistant *Mycobacterium tuberculosis* isolates in Japan. Clin Microbiol Infect. 2010; 16(8):1164-8.
6. Takiff HE, Salazar L, Guerrero C, Philipp W, Huang WM, Kreiswirth B, et al. Cloning and nucleotide sequence of *Mycobacterium tuberculosis gyrA* and *gyrB* genes and detection of quinolone resistance mutations. Antimicrob Agents Chemother. 1994; 38(4):773-80.
7. Nair J, Rouse DA, Bai GH, Morris SL. The *rpsL* gene and streptomycin resistance in single and multiple drug-resistant strains of *Mycobacterium tuberculosis*. Mol Microbiol. 1993; 10(3):521-7.
8. Caws M, Duy PM, Tho DQ, Lan NT, Hoa DV, Farrar J. Mutations prevalent among rifampin- and isoniazid-resistant *Mycobacterium tuberculosis* isolates from a hospital in Vietnam. J Clin Microbiol. 2006; 44(7):2333-7.
